# Phenotypic Evidence for the Reclassification of the *RYR1* p.(Glu4410Asp) Variant: From Uncertain Significance to Likely Benign

**DOI:** 10.3390/genes17101290

**Published:** 2026-10-15

**Authors:** Isabella Mullally, Eugene Smith, Peter Bedocs, Thomas Pranikoff, Leslie G. Biesecker, Glen A. Cook, Robert Vietor, Alakesh Bera

**Affiliations:** 1 Department of Anesthesiology, School of Medicine, Uniformed Services University of the Health Sciences, 4301 Jones Bridge Rd., Bethesda, MD 20814, USArobert.vietor@usuhs.edu (R.V.); 2 Defense and Veterans Consortium for Integrative Pain Management, Henry M Jackson Foundation for the Advancement of Military Medicine, Inc., Bethesda, MD 20817, USA; 3 Brenner Children’s Hospital, Wake Forest Baptist Medical Center, Medical Center Boulevard, Winston-Salem, NC 27157, USA; 4 Center for Precision Health Research, National Human Genome Research Institute, National Institutes of Health, Bethesda, MD 20892, USA; 5 Department of Neurology, Uniformed Services University of the Health Sciences, 4301 Jones Bridge Rd., Bethesda, MD 20814, USA

**Keywords:** *RYR1* variant, malignant hyperthermia, malignant hyperthermia susceptibility, variant reclassification, caffeine–halothane contracture test (CHCT)

## Abstract

Malignant Hyperthermia Susceptibility (MHS) is most commonly caused by pathogenic variants in the *RYR1* gene. The proliferation of *RYR1* Variants of Uncertain Significance (VUS) on diagnostic genetic panels poses a significant clinical challenge, often leading to unnecessarily complex clinical concerns. We report the case of a 27-year-old African American female with a personal history of recurrent exertional rhabdomyolysis found to have a heterozygous *RYR1* c.13230G>C p.(Glu4410Asp) variant. She later tested negative via the gold-standard Caffein–Halothane Contracture Test (CHCT). A negative CHCT test with an in silico Rare Exome Variant Ensemble Learner (REVEL) score of 0.171 meets the ClinGen MHS Variant Curation Expert Panel (VCEP) criteria for reclassification of the variant to Likely Benign. Additionally, the high allele frequency isolated within the African American (AA) population suggests a benign, ancestry-specific polymorphism maintained within the AA population rather than a pathogenic MHS mutation. Therefore, reclassifying the variant to Likely Benign prevents unnecessary muscle biopsies and alterations to perioperative anesthetic management and reduces the need for family cascade testing, and reduces use of potentially suboptimal alternative anesthetic protocols.

## 1. Introduction

Malignant Hyperthermia (MH) is a life-threatening, pharmacogenetic disorder of excitation-contraction coupling triggered by volatile anesthetic agents, such as halothane, sevoflurane, and isoflurane, and the depolarizing muscle relaxant succinylcholine [1,2]. In individuals with Malignant Hyperthermia Susceptibility (MHS), exposure to these triggering agents induces an unmodulated, sustained release of calcium through dysfunctional channels [2,3]. This sudden intracellular surge creates a hypermetabolic state that overwhelms cellular pathways. The symptoms of MH are driven by accelerating ATP consumption, enhanced glycogenolysis, cellular acidosis, and massive heat production. If left untreated, this cascading pathway rapidly manifests clinically as severe hypercapnia, generalized muscle rigidity, hyperthermia, rhabdomyolysis, metabolic acidosis, and potentially fatal cardiac arrhythmias [2,3]. Pathogenic variants in the ryanodine receptor 1 gene (*RYR1*) on chromosome 19q13.2, which encodes the primary calcium release channel of skeletal muscle. While pathogenic variants in RYR1 account for the vast majority of MHS cases, variants in CACNA1S and STAC3 also contribute to susceptibility [2].

Diagnostic confirmation relies on either the presence of a known pathogenic or likely pathogenic variant for MHS in *RYR1* or the functional evidence provided by the gold-standard in vitro Caffeine–Halothane Contracture Test (CHCT) [4,5,6]. The CHCT requires a viable skeletal muscle biopsy to evaluate the contracture response of the tissue when exposed to incremental doses of caffeine and halothane. Successfully coupling genetic testing with an overlapping negative CHCT phenotype provides the definitive functional data required to challenge predictive variant modeling and safely rule out susceptibility.

A major challenge in contemporary precision medicine is the proliferation of variants of uncertain significance (VUS) generated by expansive diagnostic panels. When a novel or rare missense change, such as *RYR1* p.(Glu4410Asp), is discovered, it is frequently classified as a VUS due to a lack of historical clinical data, even when in silico predictors lean benign. This ambiguous classification (VUS) creates a significant clinical dilemma, driving expensive medical over-management, inducing patient anxiety, and mandating alternative, potentially less optimal anesthetic plans. Furthermore, for specific patient cohorts, such as active-duty military personnel or specialized professionals, an unresolved MHS-VUS status can directly restrict operational readiness, halt deployment eligibility, and disrupt career longevity [5,6,7,8]. The propagation of VUS imposes a heavy burden on healthcare systems through increased costs, defensive over-management, and patient anxiety. Furthermore, this ambiguous classification mandates expensive diagnostic workups, potentially suboptimal anesthetic planning, and restricted operational readiness for specialized cohorts like active-duty military personnel [5,9].

**Figure 1:**
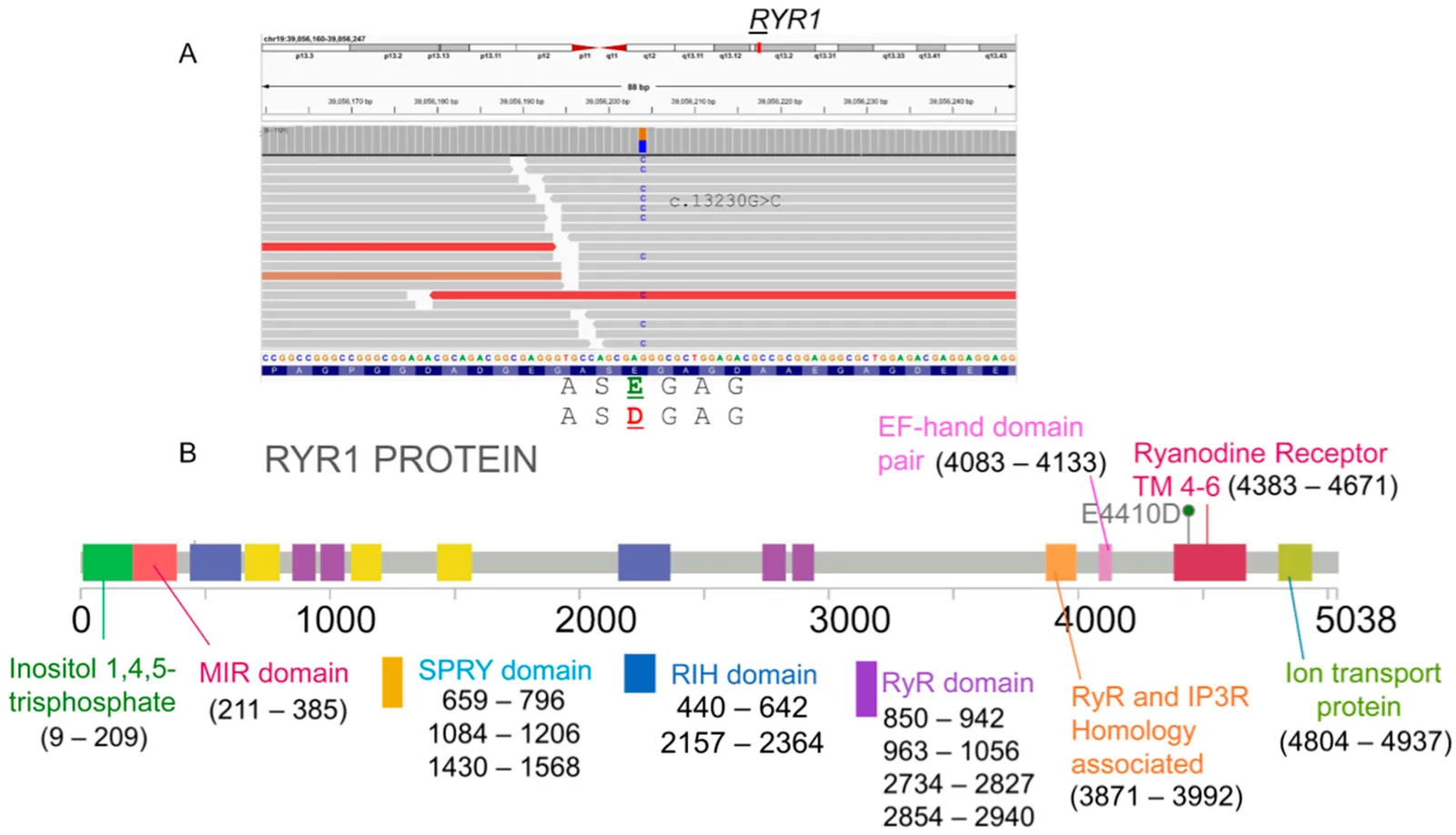
Genomic and structural localization of the heterozygous *RYR1* c.13230G>C p.(Glu4410Asp) variant. (**A**) Next-generation sequencing (NGS) alignment map showing the single-nucleotide missense substitution on Chromosome 19. (**B**) Schematic of the *RYR1* transcript flanking amino acid residue 4410. The lollipop diagram (green pin) indicates that the wild-type glutamic acid (E) residue is predicted to be changed to an aspartic acid (D) via a c.13230G>C nucleotide transversion.

To address this diagnostic bottleneck, we present the clinical case and functional evaluation of an individual harboring the heterozygous *RYR1* c.13230G>C p.(Glu4410Asp) variant in the *RYR1* gene (Figure 1). By compiling her negative CHCT data and all available criteria of pathogenicity, we aimed to clarify her risk for MHS and potentially reclassify the pathogenicity of the variant.

## 2. Method

As we are presenting a case report, the detailed experimental procedures are condensed. We have followed the protocols based on Malignant Hyperthermia Association of the U.S. (MHAUS) guidelines as previously described [1,2,6]. Briefly, diagnostic testing was conducted at the Malignant Hyperthermia Diagnostic Laboratory at the Uniformed Services University of the Health Sciences (USUHS) by qualified board-certified anesthesiologists and expert MH diagnostic personnel. Viable skeletal muscle fascicles obtained via muscle biopsy were mounted to force transducers to evaluate contracture responses [1,3,6]. The halothane challenge evaluated baseline contracture force during exposure to continuous 3.0% halothane, where a tension increase of >0.2–0.7 g after exposure, or the development of ≥0.3 g tension at 2 mM caffeine, was recorded [1,2,3]. The caffeine challenge utilized incremental concentration steps (0.5, 1.0, 2.0, 4.0, 8.0, and 32.0 mM), where a contracture force of ≥0.3 at or below the diagnostic 2.0 mM threshold indicates MHS [1,3]. Muscle tissue viability was validated at the conclusion of the protocol by verifying a robust contracture response (≥5.0 g) upon exposure to non-diagnostic 32.0 mM caffeine saturation [1,3].

## 3. Case Description

A 27-year-old African American female (IRB# ANE-08-3397; Uniformed Services University; approval date: 13 April 2026, valid until 22 April 2027) presented with a history of three episodes of exertional rhabdomyolysis over a three-year period, characterized by painful upper extremity tightness and an inability to move her arms. Each episode was precipitated by strenuous exercise. In addition to the exertional rhabdomyolysis, she experienced isolated episodes of muscle pain and swelling restricted to her arms. Her constellation of symptoms prompted a comprehensive evaluation for underlying metabolic and neuromuscular disorders. Her physical examination and electromyography (EMG) did not show any electrodiagnostic or clinical evidence of myopathy. Besides, continuous perioperative electrocardiographic (ECG) monitoring during the muscle biopsy procedure revealed no cardiac or physiological abnormalities. Given the recurrent nature of the rhabdomyolysis, genetic testing for neuromuscular disorders was performed, and the patient was referred for a muscle biopsy to undergo diagnostic CHCT.

## 4. Results

Genetic analysis was performed using the Invitae Comprehensive Neuromuscular Disorders Panel (Invitae is part of LabCorp Genetics, San Francisco, CA 94103, USA), which evaluated 211 genes including *RYR1* (NM_000540.2). A c.13230G>C; p.(Glu4410Asp) heterozygous missense variant was identified in *RYR1* (Figure 1, Table 1). Three other heterozygous genetic variants relating to muscular dystrophy were identified: *PLEC* c.2785G>A p.(Asp929Asn), *SCN4A* c.4930G>T p.(Val1644Leu), and *SCN4A* c.611C>T p.(Ala204Val) (Table 1). The focus of this paper will be on the heterozygous missense variant in *RYR1*.

Invitae originally classified the *RYR1* p(Glu4410Asp) variant as a VUS using Sherloc, a validated, semiquantitative, point-based interpretation system [9,10]. Based on various evidentiary domains, Sherloc assigns fixed points on independent pathogenic and benign scales. A variant becomes classified as VUS when it accumulates fewer than four pathogenic points or fewer than three benign points, indicating ambiguous evidence in both directions.

Further analysis of the *RYR1* p.(Glu4410Asp) variant within the Genome Aggregation Database (gnomAD) database identified its presence in 23/1,477,898 alleles or 738,949 individuals (Table 2). ClinVar contains an entry for this variant (Variation ID: 1419955). Notably, all these individuals (n = 23 + 1 current study) were of African/African American ancestry, and this variant was not identified in any other population. Ancestry designations were extracted from gnomAD (v4.1.2) genetic ancestry group annotations, where all 23 alleles were identified strictly within the African/African American reference cohort, with zero alleles reported across admixed or non-African populations. An extensive literature review failed to identify any case reports linking this *RYR1* variant to a clinical presentation of MH or a positive CHCT. Furthermore, the variant is absent from the curated list of 335 *RYR1* variants compiled by the ClinGen MHS Variant Curation Expert Panel (VCEP) [11,12,13]. In summary, the available evidence from ClinVar, Invatie, and gnomAD is currently insufficient to determine the role of this variant in disease. Therefore, it has been classified as a Variant of Uncertain Significance (VUS).

The variant was reclassified for pathogenicity using the ClinGen MHS VCEP recommendations [11,12]. The groupmax filtering allele frequency in African/African American individuals in gnomAD (0.00028) is just below the 0.0008 threshold that would meet the BS1 strong benignity criterion. The in silico meta-predictor tool, REVEL, calculated a score of 0.171 for this variant. This falls well below the established threshold of 0.5, providing supporting evidence against pathogenicity (Table 2), garnering the BP4 criterion [14,15]. We identified no other relevant criteria for this variant before testing this patient and therefore classified it as a VUS for *RYR1*-related malignant hyperthermia susceptibility.

**Figure 2:**
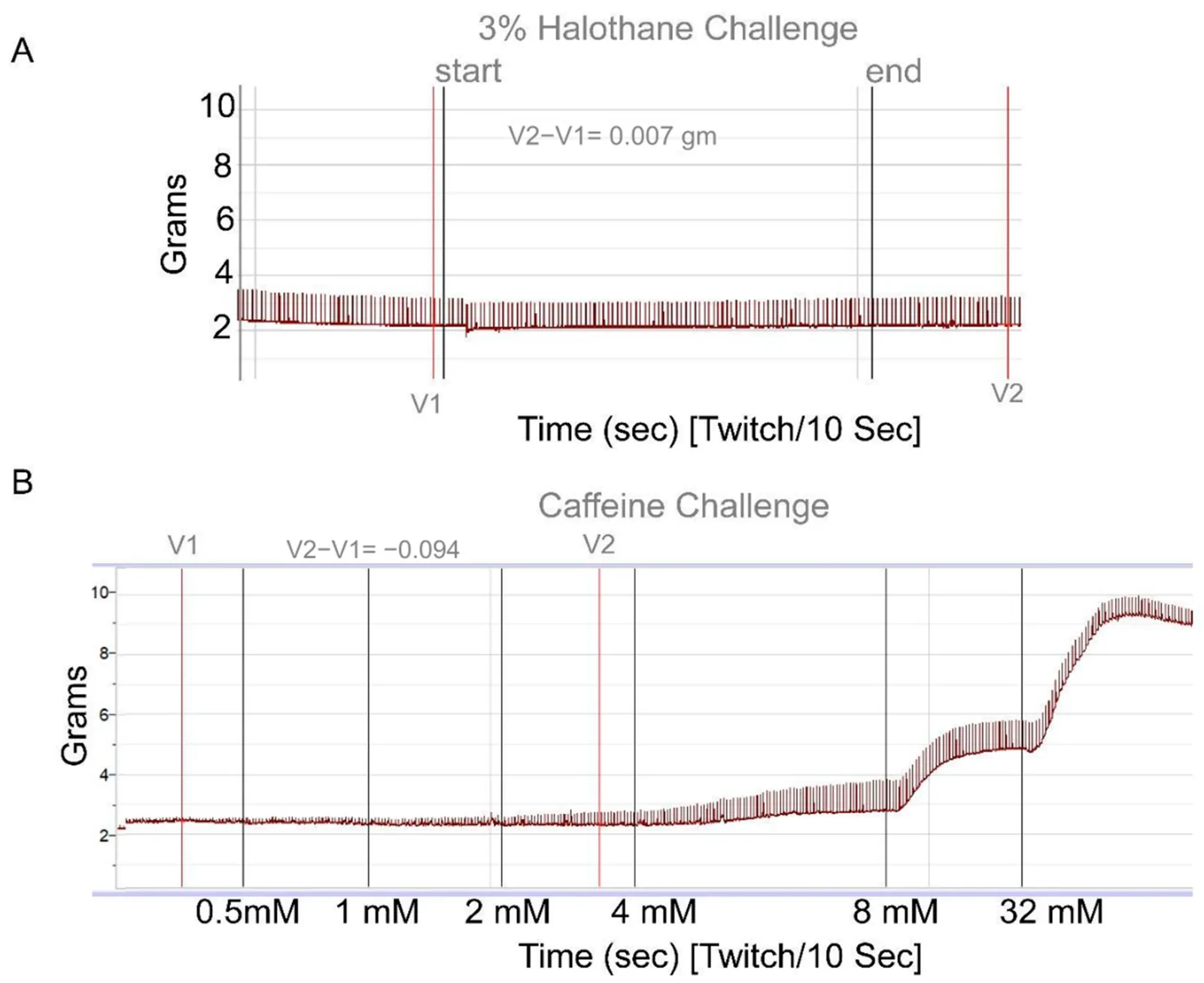
In vitro *diagnostic Caffeine–Halothane Contracture Test (CHCT) profile.* (**A**) 3% Halothane Challenge. Continuous baseline tracing showing no pathognomonic baseline contracture response during exposure to continuous 3% halothane (panel A). Vertical markers indicate the administration window (start to end of a particular Caffeine concentration). The baseline change calculation between evaluation points (V2 − V1) equals a negligible 0.007 gm, well below the diagnostic measurement (difference should be equal to or greater than 0.5 gm) required to indicate malignant contracture susceptibility. (**B**) Caffeine Challenge. Stepwise muscle pharmaco-electro-dynamic exposure profile across non-diagnostic to diagnostic (2 mM) concentrations of caffeine (0.5 mM, 1 mM, 2 mM, 4 mM, 8 mM, and 32 mM. Red vertical markers note early sampling intervals where V2 − V1 = −0.094 gm, which indicates the non-MH susceptibility within diagnostic concentrations of caffeine.

Given the VUS status and the high clinical stakes for the patient, she underwent a CHCT (Figure 2). Phenotypic evaluation showed an absence of pathognomonic baseline contractures when exposed to diagnostic thresholds of halothane or caffeine, which is a negative result. As performed according to published standards, CHCT remains the standard diagnostic test with a claimed sensitivity of 97% (95% CI, 84–100%) and a specificity of 78% (95% CI, 69–85%) [16]. Therefore, we would like to mention that patients presenting with exertional rhabdomyolysis, a negative CHCT effectively rules out classic MHS but does not exclude non-MH exertional myopathies. The presence of a negative CHCT in this individual adds criterion BS4_Mod to the classification [12], thus lowering the pathogenicity from VUS to likely benign, given that it now garners BP4 and BS4_Mod criteria and no pathogenic criteria, exceeding the “≥2 Supporting (BP1–BP7)” criteria of Richards et al. (2015) [17]. This variant would also be Bayesian classified as likely benign using the Tavtigian et al. and points-based classification adaptations [14,15,16,17,18].

## 5. Discussion

In this report, we describe a 27-year-old African American female with a personal history of recurrent exertional rhabdomyolysis who was found to have a c.13230G>C; p.(Glu4410Asp) heterozygous missense variant in *RYR1*. Population databases show that this variant is found only in individuals of African/African American ancestry. It is worth noting that among this population, this variant is present at a rate that is higher than the global population frequency of 25 of the 61 pathogenic and likely pathogenic known *RYR1*-related MHS variants. We suggest that it is unlikely that a causative variant that is this common would be unrecognized. The restriction of this variant to this demographic cohort likely points to a founder effect or an ancestral variant that arose in a specific sub-population in Africa and was maintained through genetic drift.

The clinical diagnostic evaluation yielded a negative CHCT, establishing a high likelihood of a non-MHS phenotype in this patient. While commercial clinical testing originally assigned a VUS classification to this variant, applying the ClinGen MHS VCEP recommendations with the addition of the negative CHCT supports a downgraded classification to Likely Benign [11,12].

The REVEL (Rare Exome Variant Ensemble Learner) is an ensemble in silico meta-predictor that integrates performance metrics from 13 individual computational tools. This tool has a receiver operating characteristic area under the curve (ROC-AUC) > 0.90, outperforming individual single-algorithm predictors. In the context of MHS, the ClinGen MHS VCEP specifically evaluated and calibrated REVEL thresholds against known MHS-causing variants. The ClinGen VCEP established that a REVEL score threshold of ≤0.50 accurately predicts non-pathogenicity for RYR1 missense changes [11,12,13]. Therefore, a negative contracture test in this patient provides moderate-level evidence for benignity, satisfying the BS2_Moderate criterion (Table 3). Furthermore, the REVEL score of 0.171 meets the computational threshold for benignity evidence (BP4), predicting a lack of deleterious impact on the protein. It is also important to mention that a REVEL score of 0.171 satisfies the BP4 benignity criterion (≤0.5); however, computational algorithms have recognized limitations in large ion channel genes like RYR1 and are insufficient for reclassification when used in isolation. Combining BS2_Moderate and BP4 satisfies the quantitative Bayesian scoring logic established by the American College of Medical Genetics and Genomics (ACMG) and the Association for Molecular Pathology (AMP) framework. The combination of BS2_Moderate (−2 points) and BP4 (−1 point) achieves a total Bayesian score of −3 points under the ClinGen MHS VCEP points-based framework, establishing a Likely Benign classification (range: −1 to −6 points) [11,12,15,17].

The reclassification of the *RYR1* p.(Glu4410Asp) variant from VUS to Likely Benign provides real clinical benefits. The current North American approach to individuals at risk for MHS recommends that individuals with an *RYR1* VUS may still require functional contracture testing to safely guide care [17]. Reclassifying this variant directly benefits the current patient and her family and ultimately offers broader potential to prevent unnecessary invasive muscle biopsies for future patients as clinical laboratories adopt this classification. Furthermore, it reduces the burden of carrying an ambiguous genetic risk label, simplifies future perioperative planning, and avoids the unnecessary use of alternative, more expensive, or less optimal anesthetic protocols. It is important to mention that the clinical symptoms in this current patient were exclusively precipitated by strenuous exertion. However, individuals carrying this likely benign allele who remain at rest or do not engage in intense physical exercise may stay completely asymptomatic. This highlights how exertional presentations can prompt broad diagnostic gene panel testing that uncovers silent, population-specific variants, which may initially be misattributed as causative factors for exertional symptoms. Although functional CHCT phenotyping confirms the RYR1 p.(Glu4410Asp) variant is benign for MHS, the etiology of the patient’s exertional rhabdomyolysis requires ongoing evaluation. Her symptoms may instead stem from alternative non-MH factors or sodium channel dysfunction linked to her identified SCN4A variants (p.Val1644Leu and p.Ala204Val) [19]. Finally, for specialized professional cohorts like active-duty military personnel, an unresolved MHS status can restrict operational readiness, halt deployability and disrupt career longevity [5,6,7,8].

## 6. Conclusions

We report the evaluation of a 27-year-old African American female with exertional rhabdomyolysis who was found to harbor an *RYR1* (c.13230G>C; p.(Glu4410Asp) variant with subsequent non-MHS status confirmed by CHCT. This variant is currently classified as VUS in databases. However, our experimental results and computational evidence (REVEL < 0.5) meet the formal ACMG/AMP and ClinGen MHS VCEP criteria for reclassification to Likely Benign. 

Reclassifying the *RYR1* p.(Glu4410Asp) variant prevents the clinical over-management and diagnostic over-classification of MHS within minority populations. Because the genomes of African-descended individuals overall harbor more rare variants, the presence of these variants can cause clinical challenges. The clear clinical benefits of this reclassification include eliminating the need for invasive in vitro contracture testing, avoiding potentially suboptimal alternative anesthetic protocols, and removing professional or operational readiness restrictions within specialized cohorts such as the military. This case reinforces the vital role of functional phenotypic testing in resolving genetic variant classifications.

## Figures and Tables

**Table 1:** Genetic variants found in this patient.

Gene	Transcript Number	cDNA Variant	Amino Acid Change	Variant Classification
*RYR1*	NM_000540.2	c.13230G>C	p.(Glu4410Asp)	VUS
*SCN4A*	NM_000334.4	c.4930G>T	p.(Val1644Leu)	VUS
*SCN4A*	NM_000334.4	c.611C>T	p.(Ala204Val)	VUS
*PLEC*	NM_000445.4	c.2785G>A	p.(Asp929Asn)	VUS

VUS = variant of uncertain significance.

**Table 2:** Comprehensive genetic variant & computational evidence matrix. A/AA = African/African American.

Gene Name	ClinVar ID	gnomAD Ancestry & Allele Frequency	In Silico Predictive Score (REVEL)
*RYR1* c.13230G>C p.(Glu4410Asp)	1419955	0.0003 in A/AA 0.00001 overall	REVEL: 0.171
		Isolated to 23 A/AA individuals (100% of cohort alleles)	
		No rsID available	
*SCN4A* c.4930G>T p.(Val1644Leu)	2435746	0.001 overall	REVEL: 0.290
		No rsID available	
*SCN4A* c.611C>T p.(Ala204Val)	477430	0.0008 overall	REVEL: 0.250
		rs199859508	
*PLEC* c.2785G>A p.(Asp929Asn)	471559	0.002 overall	REVEL: 0.230
		rs199833127	

**Table 3:** Benignity Criteria based on ClinGen MHS [15] Variant Curation Expert Panel (VCEP) recommendations.

Criteria	Criteria Description
Strong Criteria	
BS1	Groupmax filtering allele frequency > 0.0008 (0.08%)
BS2	Two or more variant-positive individuals with a negative IVCT/CHCT test
Moderate Criteria	
BS2_Moderate	One variant-positive individual with a negative IVCT/CHCT test
BS3_Moderate	Three or more independent ex vivo studies, NO significant release of Ca^2+^ in response to agonist
Supporting Criteria	
BP2	Observed in *cis* with a pathogenic variant in any inheritance pattern
BP4	Computational evidence suggests no impact on gene or gene product, REVEL score of ≤0.5
BP7	A synonymous (silent) variant for which splicing prediction algorithms predict no impact to the splice consensus sequence nor the creation of a new splice site AND the nucleotide is not highly conserved
BS3_Supporting	Well-established functional studies show no damaging effect on protein function - No significant increased sensitivity to RYR agonist in an approved in vitro assay, Cal release measured, n ≥ 3 - One or two independent ex vivo studies, NO significant release of Cal in response to agonist - Knock-in mouse showing no MH reaction in response to RYRI agonist AND no increased sensitivity to RYR1 agonists in ex vivo tissue/cells

## Data Availability

All original data supporting the findings of this study are included in the article. Additional information is available from the corresponding authors upon reasonable request.

## References

[1] Hopkins P.M., Gupta P.K., Bilmen J.G. (2018). Malignant hyperthermia. Handb. Clin. Neurol..

[2] Rosenberg H., Pollock N., Schiemann A., Bulger T., Stowell K. (2015). Malignant hyperthermia: A review. Orphanet J. Rare Dis..

[3] Stephens J., Schiemann A.H., Roesl C., Miller D., Massey S., Pollock N., Bulger T., Stowell K. (2016). Functional analysis of RYR1 variants linked to malignant hyperthermia. Temperature.

[4] Merritt A., Booms P., Shaw M.A., Miller D.M., Daly C., Bilmen J.G., Stowell K.M., Allen P.D., Steele D.S., Hopkins P.M. (2017). Assessing the pathogenicity of RYR1 variants in malignant hyperthermia. Br. J. Anaesth..

[5] Saha A.K., Pinyavat T. (2025). Genetic Testing for Malignant Hyperthermia Susceptibility-Threading the Needle in the Haystack. Genes.

[6] Tracy E., Mistretta K., Bedocs P., Vietor R., Bera A. (2025). Clinical and Genetic Characterization of a Novel RYR1 Variant (p.Gln474His) in Malignant Hyperthermia Susceptibility. Genes.

[7] Lee M.A., McGlinch E.B., McGlinch M.C., Capacchione J.F. (2017). Malignant Hyperthermia Susceptibility and Fitness for Duty. Mil. Med..

[8] Wren P., Chun S.M., Vietor R. (2023). Malignant Hyperthermia-A Case From Camp Humphreys, South Korea, and Lessons for the Military Health System. Mil. Med..

[9] Chen E., Facio F.M., Aradhya K.W., Rojahn S., Hatchell K.E., Aguilar S., Ouyang K., Saitta S., Hanson-Kwan A.K., Capurro N.N. (2023). Rates and Classification of Variants of Uncertain Significance in Hereditary Disease Genetic Testing. JAMA Netw. Open.

[10] Nykamp K., Anderson M., Powers M., Garcia J., Herrera B., Ho Y.Y., Kobayashi Y., Patil N., Thusberg J., Westbrook M. (2017). Sherloc: A comprehensive refinement of the ACMG-AMP variant classification criteria. Genet. Med..

[11] Johnston J.J., Dirksen R.T., Girard T., Gonsalves S.G., Hopkins P.M., Riazi S., Saddic L.A., Sambuughin N., Saxena R., Stowell K. (2021). Variant curation expert panel recommendations for RYR1 pathogenicity classifications in malignant hyperthermia susceptibility. Genet. Med..

[12] Johnston J.J., Dirksen R.T., Girard T., Hopkins P.M., Kraeva N., Ognoon M., Radenbaugh K.B., Riazi S., Robinson R.L., Saddic L.A. (2022). Updated variant curation expert panel criteria and pathogenicity classifications for 251 variants for RYR1-related malignant hyperthermia susceptibility. Hum. Mol. Genet..

[13] Ioannidis N.M., Rothstein J.H., Pejaver V., Middha S., McDonnell S.K., Baheti S., Musolf A., Li Q., Holzinger E., Karyadi D. (2016). REVEL: An Ensemble Method for Predicting the Pathogenicity of Rare Missense Variants. Am. J. Hum. Genet..

[14] Tavtigian S.V., Greenblatt M.S., Harrison S.M., Nussbaum R.L., Prabhu S.A., Boucher K.M., Biesecker L.G., ClinGen Sequence Variant Interpretation Working Group (ClinGen SVI) (2018). Modeling the ACMG/AMP variant classification guidelines as a Bayesian classification framework. Genet. Med..

[15] Tavtigian S.V., Harrison S.M., Boucher K.M., Biesecker L.G. (2020). Fitting a naturally scaled point system to the ACMG/AMP variant classification guidelines. Hum. Mutat..

[16] Allen G.C., Larach M.G., Kunselman A.R. (1998). The sensitivity and specificity of the caffeine-halothane contracture test: A report from the North American Malignant Hyperthermia Registry. N. Am. Malig. Hyperth. Regist. MHAUS. Anesthesiol..

[17] Richards S., Aziz N., Bale S., Bick D., Das S., Gastier-Foster J., Grody W.W., Hegde M., Lyon E., Spector E. (2015). Standards and guidelines for the interpretation of sequence variants: A joint consensus recommendation of the American College of Medical Genetics and Genomics and the Association for Molecular Pathology. Genet. Med..

[18] Riazi S., Watt S., Plester J., Biesecker L.G. (2026). A Proposed North American Approach for Genetic Testing of Individuals at Risk for Malignant Hyperthermia. Anesthesiology.

[19] Elia N., Nault T., McMillan H.J., Graham G.E., Huang L., Cannon S.C. (2020). Myotonic Myopathy With Secondary Joint and Skeletal Anomalies From the c.2386C>G, p.L769V Mutation in SCN4A. Front. Neurol..

